# NanoNet: Rapid and accurate end-to-end nanobody modeling by deep learning

**DOI:** 10.3389/fimmu.2022.958584

**Published:** 2022-08-12

**Authors:** Tomer Cohen, Matan Halfon, Dina Schneidman-Duhovny

**Affiliations:** The Rachel and Selim Benin School of Computer Science and Engineering, The Hebrew University of Jerusalem, Jerusalem, Israel

**Keywords:** nanobody (Nb), machine-learning (ML), protein modeling, antibody, deep learning- artificial neural network

## Abstract

Antibodies are a rapidly growing class of therapeutics. Recently, single domain camelid VHH antibodies, and their recognition nanobody domain (Nb) appeared as a cost-effective highly stable alternative to full-length antibodies. There is a growing need for high-throughput epitope mapping based on accurate structural modeling of the variable domains that share a common fold and differ in the Complementarity Determining Regions (CDRs). We develop a deep learning end-to-end model, NanoNet, that given a sequence directly produces the 3D coordinates of the backbone and C*β* atoms of the entire VH domain. For the Nb test set, NanoNet achieves 3.16Å average RMSD for the most variable CDR3 loops and 2.65Å, 1.73Å for the CDR1, CDR2 loops, respectively. The accuracy for antibody VH domains is even higher: 2.38Å RMSD for CDR3 and 0.89Å, 0.96Å for the CDR1, CDR2 loops, respectively. NanoNet run times allow generation of ∼1M nanobody structures in less than 4 hours on a standard CPU computer enabling high-throughput structure modeling. NanoNet is available at GitHub: https://github.com/dina-lab3D/NanoNet

## 1 Introduction

The large and diverse repertoire of the immune receptors, including antibodies and T cell receptors (TCRs) is behind the specific antigen recognition mechanism (1). Next generation sequencing (NGS) provides a glimpse into the blood circulating repertoires. However, the antigens and the epitopes remain unidentified. Moreover, antibodies are the most rapidly growing class of human therapeutics for a range of diseases, including cancer or viral infections. Despite their successful application, there are challenges in high-throughput cost-effective manufacturing of monoclonal antibodies (mAbs), as well as intravenous administration route. Nanobodies (Nbs) are small and highly-stable fragments derived from camelid heavy chain only antibodies (2, 3). They can reach binding affinities comparable to antibodies. Nbs can be manufactured easily in microbes and administered by aerosolization (4). Rapid Nb development is possible by camelid immunization (5, 6) or synthetic design and screening (7).

Epitope characterization is an important part of therapeutic antibody (mAb or Nb) discovery. It is critical to select epitope specific sequences from a large pool of candidates. However, high-throughput experimental structural characterization of hundreds or thousands of antibody-antigen complexes remains challenging. Computational methods for modeling antibody-antigen structures from individual components frequently suffer from high false positive rate, rarely resulting in a unique solution. There are two main bottlenecks: low accuracy of antibody CDR loop modeling and antibody-antigen scoring functions.

Antibody modeling most often proceeds in two steps. First, the conserved framework region is modeled by comparative modeling. Second, the variable CDR loops are modeled using *ab initio* techniques. The CDR3 loop which is highly variable and long presents a mini folding problem. While there are existing tools for mAb and TCR modeling, including RosettaAntibody and Rosetta TCRmodel (8–10), dedicated algorithms for reliable Nb modeling are unavailable. Compared to mAbs, Nbs generally have longer CDR3 loops and are devoid of light chains, adding additional degrees of freedom for accurate loop modeling.

Recently, deep learning has been successful in addressing challenging and fundamentally important questions in structural biology, including protein folding (11–16). Moreover, deep learning was successful in predicting restraints for the mAb CDR3 heavy chain loop modeling in DeepAb (17–19). Until recently, deep learning-based algorithms used deep learning models for restraints generation, requiring an additional optimization step to generate 3D structures (11–13, 17, 18). The structure generation step is time consuming. For example, RosettaAntibody requires about 30 minutes per model, where more than 50 models are generated per single sequence. Most recent structure prediction methods, including AlphaFold2 and RosettaFold, use deep learning models for end-to-end learning, where the input is a sequence and the output is the 3D structure (15, 16, 20, 21).

Here we use deep learning for accurate end-to-end prediction of Nb structures. While our main goal is accurate Nb modeling, NanoNet can also accurately model VH domains of the antibodies and V*β* domains of TCRs. Our deep learning model accepts the sequence (Nb, mAb VH domain, or TCR V*β* domain) as an input and produces coordinates of the backbone and C*β* atoms. NanoNet improves upon existing models using direct end-to-end learning that enables the network to learn the full 3D structure without dividing the modeling problem into framework and CDRs modeling.

## 2 Results

### 2.1 Summary of the methods

**Figure 1:**
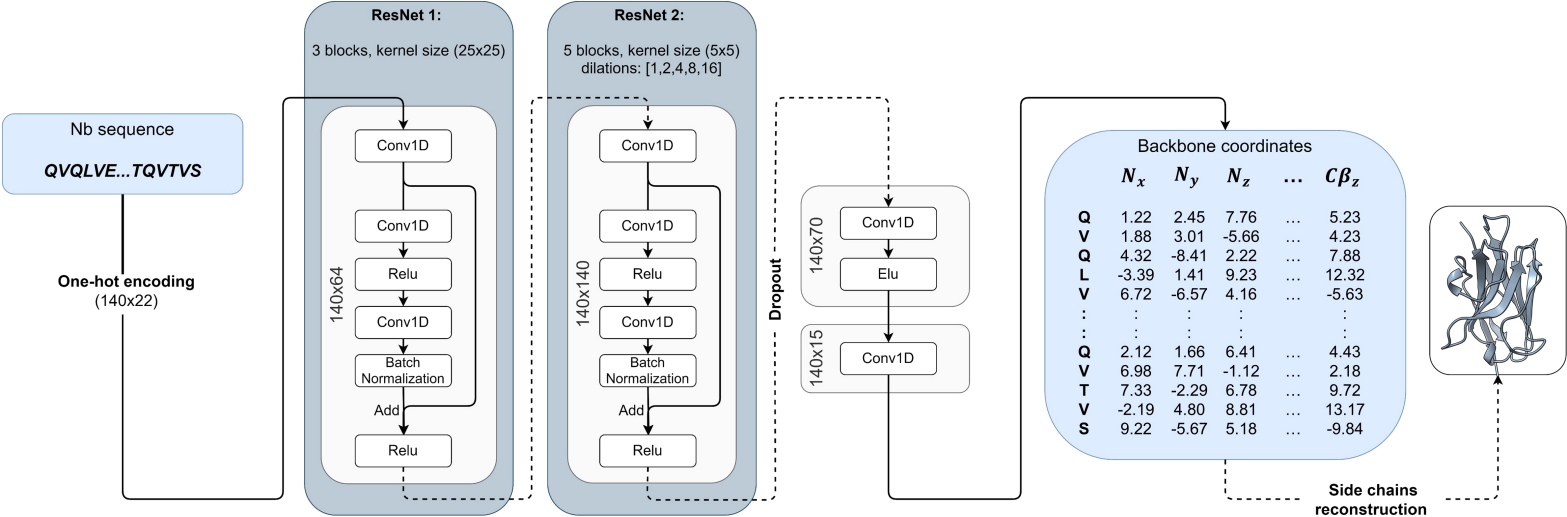
NanoNet architecture: The input of the network is the one-hot encoding of the sequence that goes into two 1D ResNets. The output is the 3D coordinates.

The input to the NanoNet is the sequence (mAb VH, Nb, or TCR V*β* domains) and the output is the backbone and C*β* coordinates for the input sequence. The network was trained on a dataset of ∼ 2,000 heavy chains of mAbs and Nb structures. The framework region of the antibodies is highly conserved with C*α* RMSD under 1Å between aligned structures. Therefore, we achieved transformational invariance for predicting 3D coordinates by aligning all the structures of the training set on a randomly selected reference structure. The VH domain structures were aligned using MultiProt algorithm with order-dependence and distance threshold of 1.4Å (22) (**Figure S1A**). This structure alignment enables the network to directly learn the VH domain 3D structure. The network is a convolutional neural network (CNN) that consists of two 1D Residual Neural Networks (ResNet) (23) (**Figure 1**). The loss is defined as an MSE (Mean Squared Error) on the backbone and C*β* coordinates, which is equivalent to the squared RMSD, and an additional term that optimizes the distance between consecutive C*α* atoms to 3.8Å. To validate NanoNet performance we used two test sets: mAb test and Nb test. mAb test consisted of 47 mAb heavy chain structures (RosettaAntibody test set (24, 25) using 99% sequence identity cutoff from the training set. The Nb test set consisted of 44 Nb structures released after July 2019 with a resolution higher than 2.5Å and sequence identity lower than 90% from the training set sequences.

### 2.2 NanoNet produces high-accuracy Nb models

**Figure 2:**
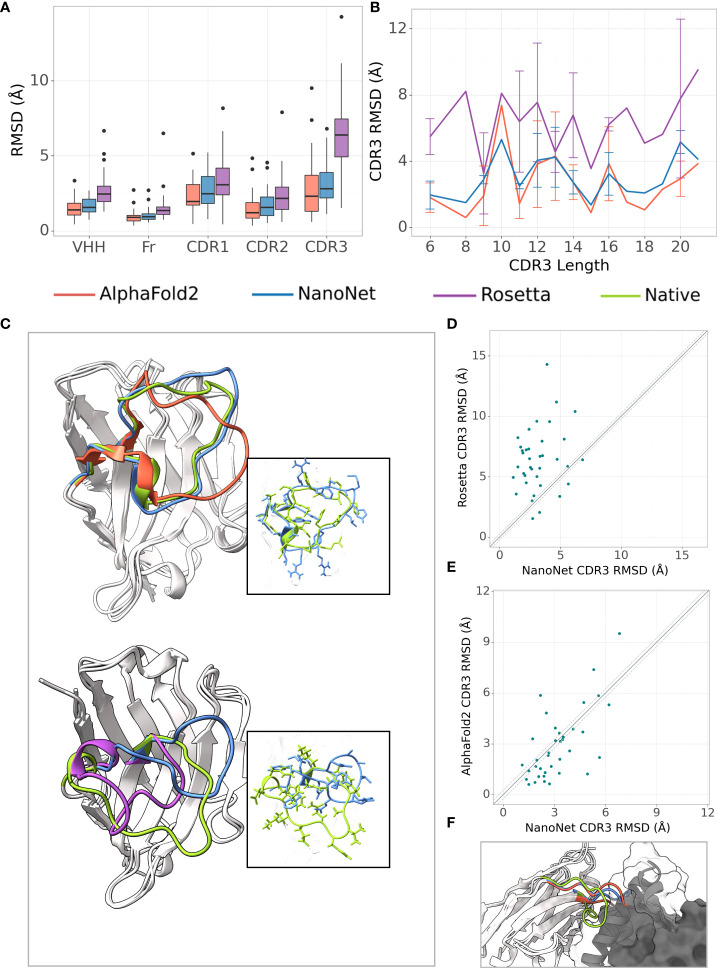
Nb modeling results. **(A)** Boxplots of RMSDs of the whole VHH region, framework, CDR1-3 loops for the Nb test set (37 Nbs), **(B)** Average RMSD of CDR3 loop as a function of loop length on the Nb test set (37 Nbs), **(C)** Test set examples of modeled structures by NanoNet (blue), RosettaAntibody (purple), AlphaFold2 (red) vs. experimental (green): PDB 6xw6 (top) - CDR3 RMSD 1.77Å, 4.06Å for NanoNet and AlphaFold2, respectively and 7n0r (bottom) - CDR3 RMSD 6.82Å,6.39A for NanoNet and RosettaAntibody, respectively, **(D)** CDR3 loop RMSD for NanoNet vs. RosettaAntibody, each dot represents a structure from the test set. The dotted line corresponds to 0.25Å RMSD, **(E)** Same as D for NanoNet vs. AlphaFold2, **(F)** Nb (PDB 6xzu) docked to its antigen, native structure –green, AlphaFold2 - red, NanoNet - blue.

The frame region average RMSD on the Nb test (44 Nbs, Methods) is 1.02Å and the median is 0.94Å (**Figures 2A**, **S3A** and **Tables 1**, **S1**). The CDR1 and CDR2 loops are also accurately modeled with average RMSDs of 2.65Å and 1.73Å (median 2.46Å and 1.54Å), respectively. For the most challenging CDR3 loop, we obtain an average RMSD of 3.16Å and a median of 2.92Å. Using the faster modeling approach (Methods) we were able to achieve an average CDR3 RMSD of 3.07Å. NanoNet obtains highly accurate models also for Nbs with longer CDR3 loops ( > 12 amino acids) (**Figure S3B**). Due to their longer length compared to mAbs (**Figure S1C**), Nb CDR3 loops often contain short 3_10_ helices. We find that NanoNet accurately reproduces such secondary structures in long CDR3 loops (**Figure 2C**, PDB 6xw6, loop length 19 residues). We also manually examine cases where NanoNet produces higher RMSD and find that often the loop conformation is correct but it’s orientation with respect to the frame is shifted (**Figure 2C**, PDB 7n0r). We noticed that we get higher RMSD values for the CDR1 and CDR2 of the Nb test set (2.65Å, 1.73Å) compared to the mAb test set (0.88Å, 0.95Å) although they do not differ much in length. This can be explained by two reasons. First, Nbs are known to form non-canonical CDR1 and CDR2 loop conformations (26, 27) which makes them harder to model compared to CDR1 and CDR2 of mAbs. The second reason is the limited number of Nbs that was used in training compared to mAbs. That said, NanoNet is still able to produce accurate CDR1 and CDR2 models (**Figure S4**). To further validate our results, we have performed 5-fold cross-validation and obtained comparable accuracy with mean CDR3 RMSD of 3.48Å with a standard deviation of 0.17Å for Nb structures (**Table S5**).

**Table 1:** Summary of mean RMSDs (Å) for the different test sets.

Nanobody test set							
Method	VHH		Fr		CDR1	CDR2	CDR3
**RosettaAntibody**	2.68±1.12		1.56±0.94		3.41±1.61	2.40±1.39	6.38±2.59
**AlphaFold2**	1.51±0.64		0.93±0.42		2.35±1.16	1.51±0.95	2.88±2.04
**AlphaFold2-No MSA**	10.37±4.78		10.06±5.01		11.30±7.71	8.02±5.15	11.06±4.51
**NanoNet+Modeller**	1.65±0.53		1.02±0.43		2.72±1.22	1.72±0.95	3.20±1.44
**NanoNet+SCWRL**	1.60±0.52		0.99±0.44		2.66±1.15	1.68±0.95	3.09±1.39
**mAb test set**							
**Method**			**Fr**		**CDR1**	**CDR2**	**CDR3**
**RosettaAntibody**			1.25±0.62		1.42±0.98	1.66±1.83	6.56±3.29
**DeepAb**			0.43±0.18		0.72±0.66	0.85±0.81	2.33±1.32
**NanoNet+Modeller**			0.64±0.18		0.88±0.67	0.95±0.82	2.38±1.17
**NanoNet+SCWRL**			0.60±0.19		0.83±0.65	0.89±0.85	2.29±1.09
**TCR test set**							
**Method**	**V** *β*		**Fr**		**CDR1**	**CDR2**	**CDR3**
**Rosetta TCRmodel**	1.49±0.68		1.07±0.60		0.93±0.33	1.24±1.29	2.79±1.28
**NanoNet+Modeller**	1.31±0.52		1.04±0.43		0.91±0.49	1.27±1.23	2.18±0.81
**NanoNet+SCWRL**	1.28±0.48		1.01±0.39		0.93±0.49	1.27±1.19	2.12±0.76
AlphaFold2*	1.48±0.58		0.89±0.19		0.99±0.61	1.19±0.63	3.07±1.45
AlphaFold2-No MSA*	15.22±5.91		15.54±6.15		18.71±9.73	12.57±4.67	11.10±5.97
NanoNet+Modeller*	1.72±0.58		1.24±0.43		1.15±0.74	2.12±1.94	2.78±0.83
NanoNet+SCWRL*	1.68±0.53		1.20±0.38		1.19±0.74	2.11±1.86	2.71±0.81

Nb test set (37 Nbs), mAb test set (47 mAbs), and TCR test set (15 TCRs). For Rosetta TCRmodel only 14 TCRs could be modeled. For AlphaFold2 only 5 TCRs were not used for training. The results for these 5 TCRs are indicated with*.

We compare our results to the results of RosettaAntibody **(Figures 2A–D**). RosettaAntibody failed to generate models for 7 Nbs from the Nb test due to the lack of suitable templates (required for CDR1 and CDR2 modeling) or program failures. For additional 6 Nbs manual CDRs definition was necessary to produce models. Overall, NanoNet has lower RMSDs for 32 out of 37 test cases (**Figure 2D**). The CDR3 RMSD is twice as low (3.20Å vs. 6.38Å), while there is also improvement in the frame region RMSD (1.02Å vs. 1.56Å), (**Table 1**).

### 2.3 Comparison to AlphaFold2

Publication of the highly accurate structure prediction model (15) prompted us to explore the accuracy of Nb structures as predicted by AlphaFold2. We used the Nb test set because it contained Nb structures published after July 2019, while AlphaFold2 was trained on structures published before August 2019. The structures in the Nb test set have less than 90*%* sequence identity to the Nbs from the training set published prior to July 2019. The run time of AlphaFold2 structure prediction for a single Nb sequence was ∼15 minutes. AlphaFold2 achieved slightly lower mean frame region RMSD (0.93Å) and mean CDR3 RMSD (2.84Å) compared to 1.02Å and 3.16Å for NanoNet (**Figure 2A**, **Table 1**). However, NanoNet had a significantly lower standard deviation (1.4Å vs. 2.0Å). The higher AlphaFold2 accuracy is most likely due to the use of the multiple sequence alignment that contained at least 3,000 sequences and training on the entire set of PDB structures. Indeed, AlphaFold2 accuracy without multiple sequence alignment, was significantly lower with RMSD values of ∼ 10.0Å (**Table 1**). In most cases NanoNet and AlphaFold2 produce relatively similar CDR3 conformations (**Figure 2C**), but we find that in some cases AlphaFold2 generated outlier CDR3 loops (**Figure 2E**). In cases where NanoNet achieved relatively high CDR3 RMSD, AlphaFold2 predictions were similar to NanoNet, however both were far from the crystal structure (**Figure 2F**). We suggest this happens due to the conformational changes upon antigen binding (28) or due to the the high conformational variability of nanobody loops. These results highlight the advantage of Nb specific structure prediction model in balancing speed and accuracy.

### 2.4 NanoNet VH models are comparable to state-of-the-art mAb modeling approaches

**Figure 3:**
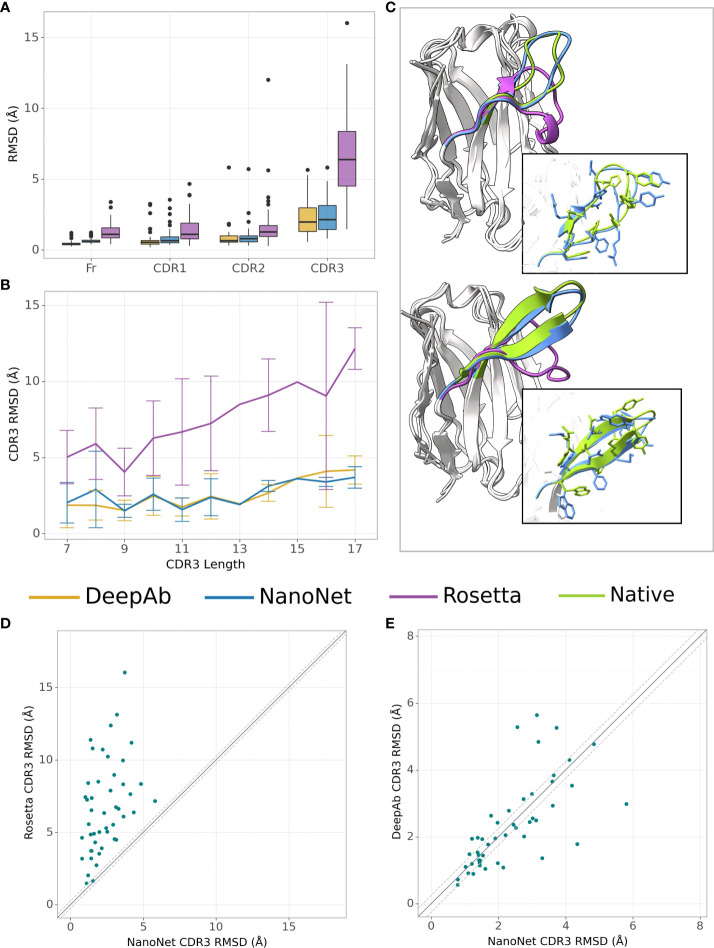
mAb VH modeling. **(A)** Boxplots of RMSDs of the framework, CDR1-3 loops for the mAbs test set (47 mAbs), **(B)** Average RMSD of CDR3 loop as a function of loop length on the mAbs test set (47 mAbs), **(C)** Test set examples of modeled structures by NanoNet (blue), RosettaAntibody (purple) vs. experimental (green): PDB 3t65 (top) and 1jfq (bottom), CDR3 RMSD 1.14Å and 1.02Å, for NanoNet, and 7.24Å and 7.42Å for RosettaAntibody, respectively, **(D)** CDR3 loop RMSD for NanoNet vs. RosettaAntibody, each dot represents a structure from the test set. The dotted line corresponds to 0.25Å RMSD, **(E)** same as D for NanoNet vs. DeepAb.

We test the method on VH modeling using the mAb test set (Methods). Overall, due to shorter average loop length and a larger number of mAbs in the training set, we obtain highly accurate models with frame region RMSD of 0.64Å overall RMSD (median 0.58Å). The CDR1, CDR2, CDR3 loops are also highly accurate with mean RMSDs of 0.88Å, 0.95Å, 2.38Å (medians 0.66Å, 0.78Å and 2.15Å), respectively (**Figures 3A, B** and **Tables 1**, **S2**). Again, we obtained from the 5-fold cross-validation comparable accuracy with mean CDR3 RMSD of 2.70Å with a standard deviation of 0.20Å for mAb structures (**Table S5**).

Similarly to Nb modeling, NanoNet VH models have significantly higher accuracy compared to RosettaAntibody**(Figures 3A–D**). NanoNet results are comparable to the accuracy reported for DeepH3 and DeepAb (17, 18) (**Figures 3A–C** and **Table 1**). Although NanoNet produced only one model per antibody vs. 50 for DeepAb, it had slightly lower number of outliers with high CDR3 RMSD (**Figure 3E**). We find that NanoNet can reproduce short secondary structure motifs in the CDR3 loops. For example, we can reconstruct the *β* turn in the CDR3 loop consisting of 14 residues (PDB 1jfq, **Figure 3C**). The main advantage of NanoNet compared to other deep learning models, such as DeepH3 or DeepAb, is that it directly produces the structure in millisecond to seconds (if side chains are added) time frame and therefore is applicable to high-throughput modeling of large databases. For comparison, loop optimization with RosettaAntibody takes at least several minutes and up to one hour per loop and normally between 500-1000 loops are generated per antibody. DeepAb takes about 10 minutes per loop and 5-10 loop conformations are usually generated per antibody.

### 2.5 Effect of sequence identity cut-off used for train and test set separation

**Figure 4:**
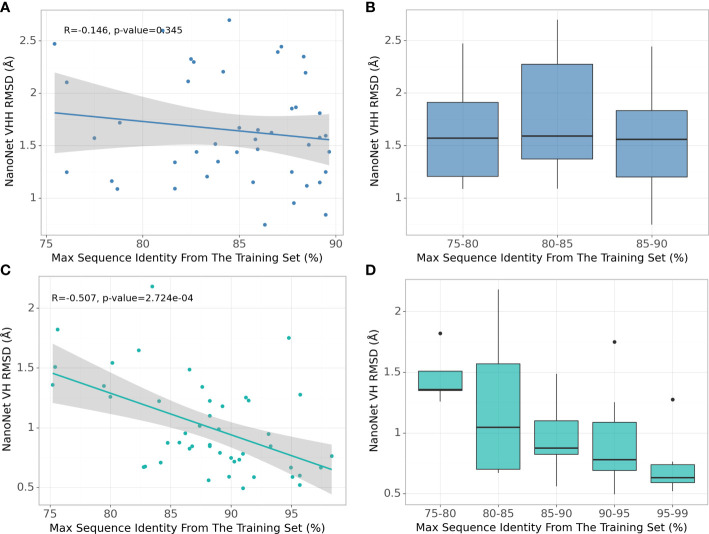
Effect of splitting the train and test sets using different sequence identity cutoffs. **(A)** NanoNet test set performance (measured as VH/VHH RMSD) as a function of maximal sequence identity to the train set, each dot represents a structure from the Nb test set (44 structures), **(B)** Test set performance with boxplots for sequence identity ranges for the Nb test set (44 structures), **(C)** same as **(A)** but for the mAb test set (47 structures), **(D)** same as **(B)** but for the mAb test set (47 structures).

Due to high baseline sequence identity between the antibodies (minimum 75% sequence identity, average 88% for mAbs and 84% for Nbs), splitting the input structures into train and test sets needs to be done carefully to prevent overfitting. To obtain the most optimal split cut-off, we compare the performance of NanoNet for each test set case as a function of maximal sequence identity (MSI) in the training set. We find that for the mAb test there is a significant negative correlation of RMSD with the training set MSI (**Figures 4C, D**). This can be explained by the high split cut-off of 99% for the RosettaAntibody dataset that was also used for training of DeepH3 and DeepAb models. In contrast, for the Nb test that was generated using the 90% cut-off, there is no correlation between RMSD and MSI (**Figures 4A, B**). Similar results were obtained for TCRs (**Figures S2C, D**).

### 2.6 Retraining for TCR V*β* modeling

The PDB contains only 200 non-redundant TCR structures (using 99% sequence identity cut-off), a number that is too low for training a deep network. Because the TCRs are structurally similar to the antibodies (**Figure S6E**), we tested if transfer learning is applicable by re-training the NanoNet on the TCR structures dataset. Our test set contains 15 structures of variable CDR3 length (**Figure S2A**). Despite the small number of available structures, accurate TCR models can be predicted for the frame and CDR loops. For the 15 structures we obtained a mean and median frame region RMSD values of 1.04Å, 0.88Å and mean RMSD values of 0.91Å, 1.27Å, 2.18Å for CDR1, CDR2 and CDR3 (medians 0.73Å, 0.87Å and 1.83Å), respectively (**Figure S6C**, **Table S3**). We compared our results to those of Rosetta TCRmodel (10), that relies on homology modeling (**Table 1**). Rosetta failed to produce a structure for one TCR due to lack of templates (PDB 6lir). While the frame, CDR1, and CDR2 RMSDs were similar, CDR3 accuracy was lower with mean RMSD of 2.79Å (**Figures S6A, C, D**). We compared to AlphaFold2 using only 5 out of 15 structures, as the remaining 10 were in AlphaFold training set (**Table 1**). AlphaFold2 produced TCR models with slightly higher accuracy of the frame and CDR1, significantly higher accuracy of CDR2 (1.19Å for AlphaFold2 vs. 2.12Å for NanoNet), and lower accuracy of CDR3 (3.07Å for AlphaFold2 vs. 2.78Å for NanoNet) (**Figures S6B, D**). We suggest that AlphaFold2 performs better in frame, CDR1, and CDR2 because of the additional information from MSA (**Table 1**). However this test set is too small for significant assessment. The accuracy of NanoNet will improve upon availability of additional TCR structures.

### 2.7 Antigen-Nb docking with NanoNet models

To further test the performance of NanoNet in the context of epitope prediction, we docked the modeled Nbs from the test set to the antigens with known antigen-Nb structures using PatchDock antibody-antigen protocol (29, 30). Antigen-Nb docking is highly challenging because docking algorithms have difficulties in sampling docked configurations with inaccurate CDR loops conformations. Additional difficulty is ranking the correct models among top-scoring ones. Here we tested whether the accuracy of NanoNet Nb models is sufficient to enable sampling of the correct orientation. Docking was applied for 17 Nbs (7 Nbs that interact with SARS-CoV-2 spike RBD (31–35), 2 Nbs that bind SARS-CoV-2 N protein (36), 1 Nb binding to SARS-CoV-1 spike RBD, 1 Nb binding to the Ebola RNA methyltransferase (37), and 4 MNV capsid protein P-domain Nbs (38). In addition, we docked two high affinity SARS-CoV-2 RBD Nbs (Nb21, Nb105) that bind to different epitopes (39). PatchDock was able to generate an acceptable quality model according to the CAPRI evaluation criteria (ligand RMSD < 10Å or interface RMSD < 4Å) for all the structures predicted by NanoNet. Overall, we achieved a mean minimal ligand RMSD of 5.42Å and a mean minimal interface RMSD of 3.58Å (**Table S4**). Medium quality models (ligand RMSD < 5Å or interface RMSD < 2Å) were sampled for 9 out of the 17 Nb structures. We have also attempted the docking of AlphaFold2 generated Nb models. Similarly, acceptable accuracy models was generated for all complexes, while medium accuracy were found for 11 out of 17 complexes. The mean minimal ligand and interface RMSDs were slightly better (4.80Å and 3.23Å). These results suggests that NanoNet and AlphaFold2 generated models can be used in docking for epitope mapping. NanoNet modeling speed allows docking of large sequence libraries.

### 2.8 Correlating sequence and structure similarity using NanoNet

**Figure 5:**
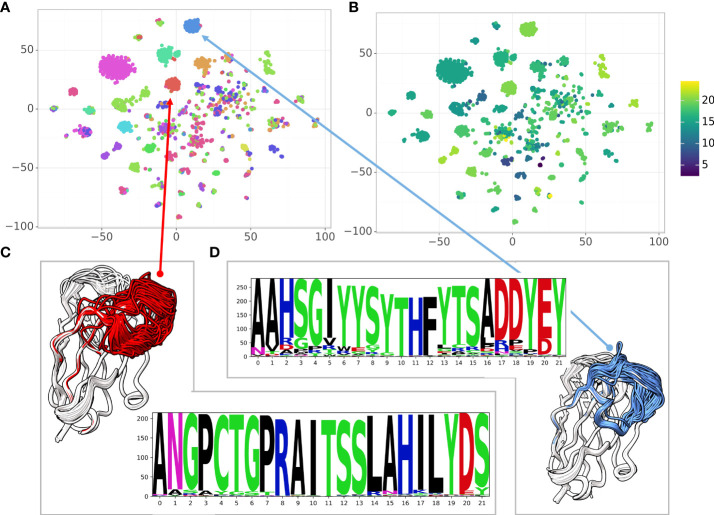
Correlating sequence and structure similarity. **(A)** The t-SNE sequence embedding colored by the structural cluster number (12 clusters), **(B)** The t-SNE sequence embedding colored by the CDR3 length, **(C, D)** Examples of structural clusters and their multiple sequence alignment, CDR3 is colored by the cluster color in the t-SNE sequence embedding.

When analyzing large antibody repertoires the main challenge is to group the sequences into clusters based on the antigen and the specific epitope the antibody binds to. While sequence similarity indicates structural similarity and similarity in the epitope, structural similarity is an additional indicator of functional similarity (40). In the case of antibodies, the sequence identity is a-priori high ( ∼ 85%). A single mutation in the CDR regions can affect loop conformations significantly and vice versa: some mutations may not affect the structure at all. Here, we used a large dataset of high affinity Glutathione S-transferase (GST) binding Nbs (6) to explore the sequence-structure similarity relationship. The dataset contains 6,222 sequences with 1,476 distinct CDR3s and 2,566 distinct CDR1-3 combinations. Because the Nbs were obtained from a single llama using a novel proteomic platform (6), it contains diverse clusters of Nbs with high sequence similarity within the cluster. We used NanoNet to model the backbone of all the 6,222 Nbs in a matter of seconds. We applied 2D dimensionality reduction of the Nbs sequences using t-SNE (41). We also clustered the 3D Nb models by their structural similarity (Methods). To analyze sequence-structure relationship between the Nbs, we colored each sequence in the t-SNE sequence embedding by the structural cluster number (**Figure 5A**) as well as the CDR3 length (**Figure 5B**). We find that the structural clusters map well onto the sequence clusters. The clusters contain tens to hundreds Nb sequences and most likely represent a combination of Nb sequences from different germlines and the affinity maturation process where residue substitutions are made to improve stability and antigen binding affinity while the Nb structure is maintained (**Figures 5C, D**). We also find several outlier Nbs that are far from their structural cluster in the sequence space, indicating that the specific mutations may change the Nb conformation. The correlation between structure and sequence clustering is independent of CDR3 length (**Figure 5B**).

## 3 Discussion

We have developed a high-throughput accurate end-to-end deep learning-based method for 3D Nb modeling. Compared to previous antibody modeling approaches, such as RosettaAntibody or DeepAb, the method directly predicts the 3D coordinates of the backbone and C*β* atoms for the whole Nb sequence without separate modeling of frame and CDR regions. Because NanoNet was trained on antibody VH domain structures (as well as Nbs), it has high accuracy in VH modeling. The accuracy of the method is significantly higher than standard loop modeling methods and comparable to deep learning based approaches, including AlphaFold2 (**Figure 2**, **Figure 3**). Moreover, the run times are significantly lower, enabling high-throughput applications. Availability of several tools for antibody structure prediction, enables to generate several CDR loops conformations for downstream applications, such as docking, increasing its accuracy. We have also compared NanoNet to the recently proposed IgFold model (42). IgFold has slightly higher accuracy (mean CDR3 RMSD is lower by 0.3Å). This can be explained by a larger training set as well as the use of antibody-specific language models. In the future work, we will test whether the use of a multiple sequence alignment, or a language model pre-trained on antibody sequences, can further improve NanoNet accuracy. It is important to understand that there is a upper bound on the accuracy of antibody modeling tools, due to the limited number of solved structures, the noisy data and off course the fact that the CDR loops are flexible and can change conformation upon binding to an antigen. Structural alignment of the VH domains enabled us to use a relatively simple model and data representation. Because there are only ∼ 2,000 antibody structures available, more complex models with larger numbers of parameters might lead to data overfitting. We find that despite longer CDR3 loops of the Nbs, NanoNet can model them with high accuracy, reproducing the short secondary structures, such as 3_10_ helix or beta-turn (**Figures 2**, **3**). We extend the approach to accurate TCR V*β* modeling by transfer learning and train a model using order of magnitude less structures (less than 200 TCRs vs. more than 2,000 mAbs and Nbs).

The high modeling speed of NanoNet (a few milliseconds per Nb), enables accurate modeling of entire antibody repertoires from NGS experiments. This modeling can further enable analysis of the serum repertoire according to antigen specificity (6). We applied the method for modeling a large dataset of GST Nb sequences obtained from a single animal (6) and found that sequence similarity often correlates with structural similarity (**Figure 5**). In addition, we found that the NanoNet loop accuracy enables accurate antigen-Nb docking for epitope mapping.

The current NanoNet implementation is trained to predict only backbone and C*β* coordinates. Representation of side chains (for example using center of mass) in the NanoNet, as well as refinement using GNN-based deep-learning approaches can further improve prediction accuracy (43). Our approach can be extended for modeling the whole mAb structure (light and heavy chains) by training a similar network for the light chain prediction and combining the light and heavy chains using most similar template structure (44, 45). We expect that NanoNet will be applicable in therapeutic applications that require epitope mapping, as well as studies that perform serum repertoire studies. In the future, the network can be extended towards design of novel sequences with high stability and specificity for antigens of interest (46).

## 4 Methods

### 4.1 Network architecture

The sequences are represented by an input tensor of 140x22, where 140 represents the maximal length of the heavy chain and the 22 channels are used for one-hot encoding of the 20 amino acids (one channel for unknown amino acid and one for insertion). The tensor is padded with insertion values for all sequences shorter than 140. The network consists of two 1D ResNets (23) (**Figure 1**). The first ResNet has a relatively big kernel size of 25 to enable the network to learn the frame and CDR loops. Next, we convert the tensors to 140x140 dimension using a 1D convolution with 140 kernels. This second ResNet captures the inter-residue interactions and consists of kernels of size 5 with dilated convolutions (47). We use five different dilation values (1, 2, 4, 8, and 16). Finally, we convert the tensors to 140x70 and then to 140x15. This last tensor represents the coordinates of the N, C*α* , C, O, and C*β* atoms and is compared directly to the actual coordinates to calculate loss. The whole network consists of ∼ 2,000,000 parameters. We add a dropout layer of 25% after the second ResNet to prevent overfitting. The network was implemented using the TensorFlow library with keras (48).

### 4.2 Training

The training was performed using ADAM optimizer (49) with a learning rate of 0.001, batch size of 16 and ∼ 130 epochs using a model checkpoint on the validation loss (**Figure S1B**). The training took less than 10 minutes on a GeForce RTX 2080 Ti. The 5-fold cross validation was performed by splitting all the data to training and validation sets such that the validation set contained 100 mAb structures and 50 Nb structures. After training we calculated the RMSD of each structure in the validation set and reported the mean RMSD for each of the 5 repetitions.

### 4.3 Loss function

We used a loss function comprised of two terms: the first term is a MSE loss between the predicted backbone and C*β* coordinates and the experimental coordinates after alignment to a reference structure, this is equivalent to the RMSD. The second term is a loss that optimizes the distance between consecutive C*α* atoms to 3.8Å and defined as (for a sequence of length N):


(1) $$L_{C\alpha }\left(\hat{y}\right)=\sum_{i=1}^{N-1}{\left[\;d\left(C\alpha_{i},C\alpha_{i+1}\right)-3.8\;\right]}^{2}$$

Where *Cα_i_* are the 3D coordinates of the *Cα* atom in the *i* amino acid of the predicted structure, and *d* is Euclidean distance. The total loss was defined as the sum of two terms: 


(2) $$L\left(y,\hat{y}\right)=MSE\left(y,\hat{y}\right)+L_{C\alpha }\left(\hat{y}\right)$$

### 4.4 Structure prediction

The prediction of the backbone and C*β* coordinates based on the trained network is straightforward and takes about 6 milliseconds on a GPU or about 20 milliseconds on a CPU. The full atom model is obtained using Modeller (50, 51) that also optimizes bond lengths and angles. NanoNet produces a single structural model for each input sequence. The faster modeling protocol uses SCWRL (52) to reconstruct the side chains without the optimization step and does not modify the coordinates of the backbone atoms. The running times for 1,000 structures on a standard CPU were as follows: only backbone + C*β* - less than 15 seconds, backbone + SCWRL - about 20 minutes, backbone + Modeller - about 80 minutes.

### 4.5 Retraining for TCRs structure prediction

The NanoNet network for TCRs was trained starting from the pretrained antibody network using the TCR training set structures with the same parameters, loss function, and learning rate. It was trained for 50 epochs using model check-point on the validation loss (**Figure S2B**).

### 4.6 Dataset

Due to a relatively small number of Nb structures in the PDB, we trained our model using the heavy chains of both mAbs and Nbs. The antibody structures were obtained from abYbank/AbDb (53) and SAbDab (54, 55); a total of 2,085 non-redundant structures of Nbs (319) and mAb heavy chains (1,766) were used. We selected non-redundant structures with resolution of 4Å or better. Missing residues ( < 6 consecutive residues) were added by MODELLER v9.18 automodel protocol (50, 51). The data was split into training, testing, and validation sets. In total 1,843 structures (1590 mAb, 253 Nb) were used for training and 150 structures (129 mAb, 21 Nb) were used for validation (7.5%). In addition, we used two test sets: mAb test and Nb test. The mAb test consisted of 47 mAb heavy chain structures (RosettaAntibody test set (24, 25). The Nb test consisted of 44 Nb structures that were deposited into PDB after July 2019 with resolution higher than 2.5Å. The average CDR3 loop length was 12.0, 12.2, 10.9, and 13.3 amino acids for train, validation, mAb test, and Nb test, respectively (**Figure S1C**). We used DeepAb data separation to enable direct comparison of mAb modeling which was divided into train and test using 99% sequence identity cutoff (17, 18). Specifically, for assessment of Nb modeling we separated the available Nb structures such that there are no sequences with sequence identity higher than 90% between the train and test sets. The TCR structure dataset with 196 structures was obtained from the STCRDab (56). The data was split into train, validation, and test sets similarly to Nb dataset, this time using a 92% sequence identity cutoff which resulted in 15 structures in the test set. For TCRs, the average CDR3 loop length was 14.0 and 14.5 amino acids for train and test, respectively (**Figure S2A**).

### 4.7 Evaluation

We evaluate the accuracy of the predicted models using the backbone RMSD (N, C*α* , C, O atoms) calculated on the whole VH structure, as well as the RMSD of the frame and the three CDR loops. The RMSD of the loops was calculated based on the superposition of the frame regions that minimizes the backbone RMSD (57). The CDRs were defined using the Chothia numbering scheme and definition (58).

### 4.8 Comparison to other methods

We used the recommended RosettaAntibody protocol for mAb heavy chains and for Nbs (8, 25). Identical structures were excluded from RosettaAntibody template search. In total, 100 loops were generated and the best-scoring was selected for comparison. We used 100 loops instead of the recommended 1,000 due to the limits of our computing resources. For Rosetta TCRmodel we used a sequence identity cutoff of 95% (Supplementary data). For DeepAb we used the same test set and compared to the reported RMSD values of the top 1 model out of 50 (18). To generate AlphaFold2 models for both the Nb and the TCR test sets we have used the ColabFold implementation without structural templates (59).

### 4.9 Docking and modeling of SARS-CoV-2 RBD nanobodies

The docking was done using PatchDock antibody-antigen protocol (29, 30) that focuses the search on the CDR loops. The results were clustered using RMSD of 4.0Å. Docking results were assessed using a standard CAPRI measures of interface RMSD and ligand RMSD (60).

### 4.10 Sequence embedding

The sequences were first aligned using ANARCI antibody numbering tool (61) that numbers the sequence based on 126 canonical positions. After the alignment, the sequences were converted into a one-hot encoding vector representation, resulting in a representation with a vector of length 2,646 for each sequence.

### 4.11 Structural distance between the Nbs

Structural similarity between a pair of Nb structures was defined by counting the number of pairs of C*α* atoms (one from each Nb structure) within a short distance ( < 1Å). Note that NanoNet predicts aligned Nb structures, therefore the number of aligned C*α* atoms is a measure of structural similarity. We define a structural distance as follows:


(3) $$Structural\;distance\left(V,W\right)=1-\frac{\#\left(aligned\;C\alpha \;atoms\right)}{min\;(\|V\|,\|W\|)}$$

We quantify the structural similarity for all pairs of Nbs and produce a distance matrix. We used the pairwise distance matrix to cluster the structures by K-Medoids algorithm (62) with k=12.

## Data availability statement

The source code, the trained model and a Colaboratory notebook for running NanoNet are available at GitHub: https://github.com/dina-lab3D/NanoNet.

## Author contributions

DS-D conceived the research and supervised the study, TC developed and benchmarked the method, MH assisted with deep learning architectures. TC MH and DS-D drafted and edited the manuscript. All authors contributed to the article and approved the submitted version.

## Funding

The reasearch was supported by ISF (1466/18) and Minerva Stiftung

## Conflict of interest

The authors declare that the research was conducted in the absence of any commercial or financial relationships that could be construed as a potential conflict of interest.

## Publisher’s note

All claims expressed in this article are solely those of the authors and do not necessarily represent those of their affiliated organizations, or those of the publisher, the editors and the reviewers. Any product that may be evaluated in this article, or claim that may be made by its manufacturer, is not guaranteed or endorsed by the publisher.
