# Supplementary material for: NanoNet: Rapid and accurate end-to-end nanobody modeling by deep learning
Source: Front Immunol. 2022 Aug 12;13:958584. doi: 10.3389/fimmu.2022.958584 (PMC9411858; doi:10.3389/fimmu.2022.958584)
Supplement: Supplementary file 1 [file DataSheet_1.pdf]

Supplementary Material

1 ROSETTA COMMANDS

We used the following command for RosettaAntibody grafting stage:

antibody.linuxgccrelease -n_multi_templates 1 -vhh_only -exclude_homologs true -fasta nb_fasta

and the following command for the H3 loop modeling using the recommended abH3.flags file:

antibody_H3.linuxgccrelease @abH3.flags -s grafting/model-0.relaxed.pdb -nstruct 100

We used the following command for Rosetta TCRmodel:

tcrmodel.linuxgccrelease -alpha <alpha_sequence> -beta <beta_sequence> -template_identity_cutoff 95 -blastp_identity_cutoff 95

2 SUPPLEMENTARY TABLES

PDB	VHH	Fr	CDR1	CDR2	CDR3	CDR3 Length	Max train identity
**6ocd**	2.70	2.70	1.31	3.01	3.06	14	0.84
**6z6v**	1.09	0.73	1.34	0.63	2.19	17	0.79
**7a4t**	1.25	1.04	2.13	1.29	1.91	12	0.89
**7c8v**	0.84	0.56	0.84	2.03	1.87	6	0.89
**6yu8**	1.44	0.89	3.48	2.26	2.19	13	0.83
**6xw5**	1.52	0.94	4.08	0.52	2.72	9	0.84
**7n0i**	1.44	1.32	1.92	0.85	1.93	17	0.85
**6waq**	1.15	0.63	1.82	0.67	2.53	16	0.89
**6xyf**	1.67	0.95	3.56	1.00	3.17	17	0.85
**7a0v**	1.34	0.73	2.77	0.98	2.82	14	0.82
**7d30**	2.35	1.12	2.93	1.98	6.20	12	0.88
**7mfv**	0.95	0.58	1.15	2.39	1.98	6	0.88
**7a50**	1.56	0.84	1.80	2.82	3.31	17	0.86
**7ldj**	2.19	1.08	4.62	1.86	4.11	21	0.88
**7kn5**	2.39	1.13	5.02	1.57	4.67	20	0.87
**6uht**	2.30	2.09	4.13	0.97	2.46	16	0.83
**7kjh**	1.62	1.03	3.73	2.58	2.68	11	0.87
**6xxo**	1.57	1.13	4.01	2.73	1.36	15	0.78
**7aqg**	1.47	0.75	1.63	3.10	3.29	12	0.86
**6x05**	2.47	1.43	3.61	2.48	5.60	13	0.75
**6xw7**	0.74	0.58	0.78	1.31	1.50	8	0.86
**6lz2**	1.12	0.60	2.27	0.69	3.35	6	0.88
**6z10**	2.60	1.34	2.59	1.15	5.65	20	0.81
**6obm**	1.85	1.20	4.16	1.21	3.49	12	0.88
**6lr7**	1.16	0.57	2.94	0.61	2.08	18	0.78
**6xzu**	1.87	1.00	1.97	1.23	5.31	10	0.88
**7mfu**	1.25	0.63	1.08	4.55	1.12	6	0.88
**6xw4**	2.33	1.21	5.23	1.50	4.94	13	0.83
**6obg**	1.65	0.90	3.59	1.02	3.53	12	0.86
**6zrv**	1.44	0.77	1.96	1.78	3.32	14	0.90
**7now**	1.58	1.05	3.35	0.87	2.64	19	0.89
**6obo**	1.51	0.77	3.38	1.00	3.30	12	0.89
**7k84**	2.20	1.03	1.15	2.10	5.20	17	0.84
**6oca**	2.11	1.19	4.18	3.10	3.02	16	0.82
**6ui1**	2.10	1.22	2.49	0.93	4.73	16	0.76
**7a48**	1.35	0.86	2.37	1.82	3.07	9	0.84
**6obc**	1.25	0.86	2.46	1.21	2.42	11	0.76
**6z1v**	1.20	0.93	1.87	0.78	2.22	14	0.83
**7kgj**	1.81	0.81	2.46	4.20	3.90	12	0.89
**6xw6**	1.09	0.79	1.66	1.46	1.77	19	0.82
**6obe**	1.72	1.24	4.12	2.02	2.39	11	0.79
**7d2z**	1.59	1.59	1.62	1.66	1.50	6	0.89
**7n0r**	2.44	1.14	1.13	2.29	6.82	12	0.87
**7lvu**	1.15	0.88	2.04	1.76	1.73	14	0.86
**Mean**	**1.65**	**1.02**	**2.65**	**1.73**	**3.16**	**13.30**	**0.85**
**STD**	**0.51**	**0.40**	**1.20**	**0.94**	**1.39**	**4.07**	**0.04**
**Median**	**1.57**	**0.94**	**2.46**	**1.54**	**2.92**	**13**	**0.86**

Table S1. NanoNet results for the Nb test set. RMSDs (Å) for the whole VHH domain, followed by frame and CDRs1-3, CDR3 length, and maximal sequence identity to a Nb in the training set.

PDB	VH	FR	CDR1	CDR2	CDR3	CDR3 Length	Max train identity
**1mtq**	0.59	0.39	0.50	0.42	1.61	9	0.90
**2r8s**	1.28	0.66	0.90	2.59	3.02	12	0.96
**3nps**	1.75	1.21	2.93	1.41	3.19	17	0.95
**2v17**	1.10	0.68	0.47	0.98	2.93	11	0.88
**1seq**	1.34	0.54	0.88	0.87	3.62	14	0.88
**1fns**	1.23	0.86	0.64	0.83	2.77	14	0.91
**3go1**	1.36	0.74	1.98	1.97	3.00	14	0.75
**2adf**	0.73	0.60	0.49	0.49	1.69	9	0.91
**1mfa**	0.67	0.55	0.56	0.76	1.41	9	0.97
**3eo9**	0.84	0.57	0.81	0.74	1.98	12	0.87
**4nzu**	1.26	0.57	1.01	0.50	3.15	16	0.80
**2e27**	1.18	0.57	1.03	0.85	4.11	7	0.89
**3gnm**	0.87	0.61	0.70	0.69	2.31	9	0.85
**2ypv**	1.22	1.08	0.43	0.85	2.45	10	0.84
**3p0y**	0.75	0.54	1.05	1.19	1.38	12	0.90
**3t65**	0.59	0.46	0.73	0.70	1.14	11	0.95
**2xwt**	1.02	0.55	1.47	0.74	2.75	10	0.87
**3umt**	0.86	0.57	0.53	0.95	2.21	10	0.88
**1dlf**	1.25	0.63	1.29	0.89	3.65	10	0.91
**3e8u**	0.67	0.59	0.42	0.57	1.42	8	0.83
**3mxw**	0.88	0.58	0.74	1.26	2.15	10	0.86
**3mlr**	1.51	0.90	0.66	0.92	3.61	15	0.75
**4h20**	0.82	0.67	0.45	0.87	1.78	10	0.87
**1gig**	1.22	0.62	1.01	0.64	3.13	14	0.88
**3liz**	1.49	0.49	0.71	0.49	4.84	10	0.87
**2vxv**	0.99	0.56	0.47	1.10	2.57	12	0.89
**3g5y**	0.52	0.49	0.60	0.44	0.80	7	0.96
**3lmj**	2.18	0.97	3.53	5.70	3.73	16	0.83
**4h0h**	0.84	0.81	0.34	0.29	1.44	10	0.88
**3oz9**	0.79	0.53	0.50	0.99	1.98	10	0.89
**3giz**	0.78	0.51	0.47	0.45	1.91	13	0.91
**1oaq**	0.67	0.49	0.62	0.54	1.52	11	0.95
**2d7t**	0.95	0.74	0.80	0.54	2.53	7	0.93
**1jfq**	0.76	0.75	0.58	0.54	1.02	12	0.98
**3hc4**	0.95	0.96	0.48	0.92	1.25	7	0.86
**1mlb**	0.60	0.48	0.47	0.78	1.45	7	0.96
**2w60**	0.59	0.47	0.39	0.39	1.39	9	0.92
**2fb4**	1.65	0.62	0.62	0.78	4.19	17	0.82
**2fbj**	0.72	0.60	0.50	0.68	1.54	9	0.90
**3vow**	0.71	0.58	0.91	1.11	1.10	11	0.84
**1jpt**	0.67	0.56	0.70	0.59	1.46	8	0.83
**4f57**	1.35	0.60	0.85	1.51	3.31	16	0.79
**3i9g**	1.54	0.69	0.93	1.24	4.35	12	0.80
**1nlb**	0.49	0.46	0.38	0.55	0.79	9	0.91
**3hnt**	0.56	0.44	0.35	0.81	1.21	9	0.88
**3m8o**	1.82	0.98	1.74	1.25	5.81	8	0.76
**4hpy**	0.85	0.49	2.52	0.50	1.21	11	0.93
**Mean**	**1.01**	**0.64**	**0.88**	**0.95**	**2.38**	**10.94**	**0.88**
**STD**	**0.39**	**0.18**	**0.67**	**0.82**	**1.17**	**2.79**	**0.06**
**Median**	**0.87**	**0.58**	**0.66**	**0.78**	**2.15**	**10**	**0.88**

Table S2. NanoNet results on the mAb test set. RMSDs (Å) for the whole VH domain, followed by frame and CDRs1-3, CDR3 length, and maximal sequence identity to a mAb VH in the training set.

PDB	Vβ	Fr	CDR1	CDR2	CDR3	CDR3 Length	Max train identity
**6vrn**	1.00	0.63	0.58	0.62	2.24	15	0.92
**6c61**	1.81	1.69	0.92	1.02	2.88	16	0.90
**2ial**	0.85	0.72	0.53	0.50	1.72	11	0.93
**6fr8**	0.80	0.66	0.42	0.74	1.49	14	0.93
**4ww1**	1.01	0.88	0.55	0.91	1.83	12	0.82
**1kgc**	0.80	0.61	0.90	0.87	1.39	13	0.81
**6ovn**	2.01	1.81	0.88	2.50	2.89	17	0.93
**4ei6**	1.61	1.75	1.24	1.32	1.13	14	0.71
**6r2l**	2.28	1.24	1.33	5.34	2.97	18	0.86
**2cdf**	1.33	0.91	0.73	0.58	2.92	15	0.93
**6frb**	0.88	0.72	1.27	0.66	1.40	12	0.64
**6lir**	1.19	1.15	0.61	0.80	1.83	14	0.58
**6ovo**	2.08	1.40	2.36	1.37	4.00	17	0.93
**3vxq**	0.89	0.78	0.64	0.68	1.58	13	0.93
**5d2n**	1.12	0.68	0.69	1.08	2.39	16	0.92
**Mean**	**1.31**	**1.04**	**0.91**	**1.27**	**2.18**	**14.47**	**0.85**
**STD**	**0.51**	**0.43**	**0.49**	**1.23**	**0.81**	**2.07**	**0.12**
**Median**	**1.12**	**0.88**	**0.73**	**0.87**	**1.83**	**14.00**	**0.92**

Table S3. NanoNet results for the TCR test set. RMSDs (Å) for the whole Vβ domain, followed by frame and CDRs1-3, CDR3 length, and maximal sequence identity to a TCR Vβ in the training set.

PDB	Antigen	Min ligand	Min interface
**6yu8**	Ebola RNA methyltransferase	9.56	4.87
**6xw5**	MNV capsid protein P-domain	4.31	2.19
**6xw7**	MNV capsid protein P-domain	3.13	1.30
**6xw4**	MNV capsid protein P-domain	6.46	3.66
**6xw6**	MNV capsid protein P-domain	5.40	2.16
**6waq**	SARS-CoV-1 RBD	4.25	2.51
**7n0i**	SARS-CoV-2 N protein	5.29	1.79
**7n0r**	SARS-CoV-2 N protein	5.28	3.11
**7c8v**	SARS-CoV-2 RBD	3.55	4.60
**7d30**	SARS-CoV-2 RBD	3.63	2.55
**7ldj**	SARS-CoV-2 RBD	4.28	4.75
**7kn5**	SARS-CoV-2 RBD	4.50	4.68
**7mfu**	SARS-CoV-2 RBD	3.45	4.30
**7kgj**	SARS-CoV-2 RBD	8.18	5.69
**7d2z**	SARS-CoV-2 RBD	7.82	4.00
**nb105**	SARS-CoV-2 RBD	7.40	3.84
**nb21**	SARS-CoV-2 RBD	5.67	4.85
**Mean**	-	**5.42**	**3.58**
**STD**	-	**1.87**	**1.30**
**Median**	-	**5.28**	**3.84**

Table S4. Docking results for NanoNet generated models. Minimal ligand and interface RMSDs (Å) for docking models generated by PatchDock.

Model	VH	Fr	CDR1	CDR2	CDR3
#1	1.24	0.77	1.20	1.18	2.72
#2	1.05	0.70	1.00	1.11	2.34
#3	1.26	0.76	1.12	1.21	2.86
#4	1.22	0.75	1.12	1.07	2.79
#5	1.20	0.71	1.18	1.15	2.77
**Mean**	**1.20**	**0.74**	**1.12**	**1.14**	**2.69**
**STD**	**0.08**	**0.03**	**0.08**	**0.06**	**0.21**

Model	VHH	Fr	CDR1	CDR2	CDR3
#1	1.61	0.90	2.29	1.50	3.42
#2	1.75	1.11	2.19	1.57	3.45
#3	1.60	0.95	2.37	1.71	3.24
#4	1.75	0.97	2.50	1.65	3.63
#5	1.77	1.11	2.19	1.53	3.66
**Mean**	**1.69**	**1.01**	**2.31**	**1.59**	**3.48**
**STD**	**0.08**	**0.10**	**0.13**	**0.09**	**0.17**

Table S5. 5-Fold Cross Validation Results. Mean RMSDs (Å) for the whole VH/VHH domain, followed by frame and CDRs1-3 for each validation split. Top - mAb (mean of 100 mAb structures), Bottom - Nb (mean of 50 Nb structures) .

3 SUPPLEMENTARY FIGURES

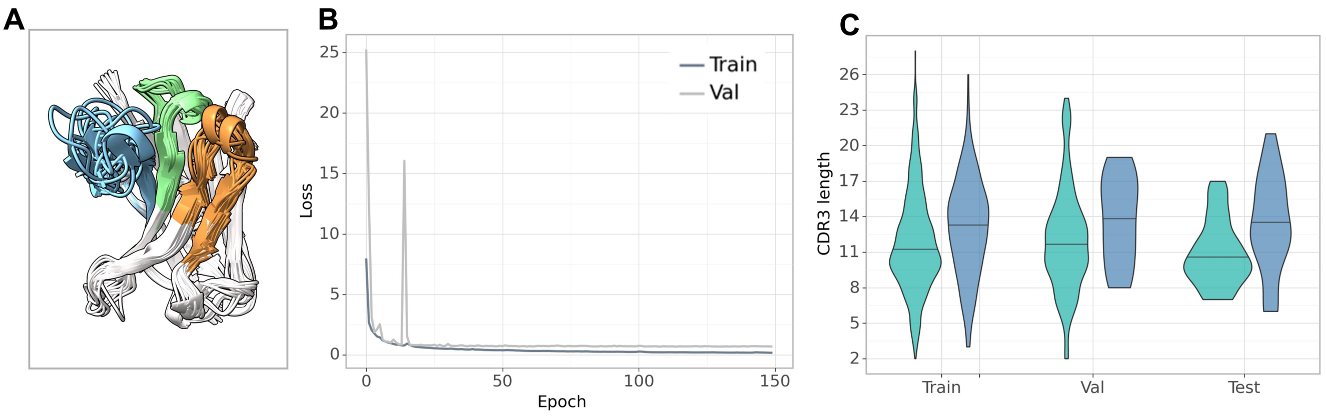

Fig. S1. **(A)** Structural alignment of the antibodies from the training set prior to coordinate extraction for training the network, **(B)** Training and validation loss during the training process, **(C)** CDR3 lengths of the training, validation, and test sets (mAb green, Nb blue).

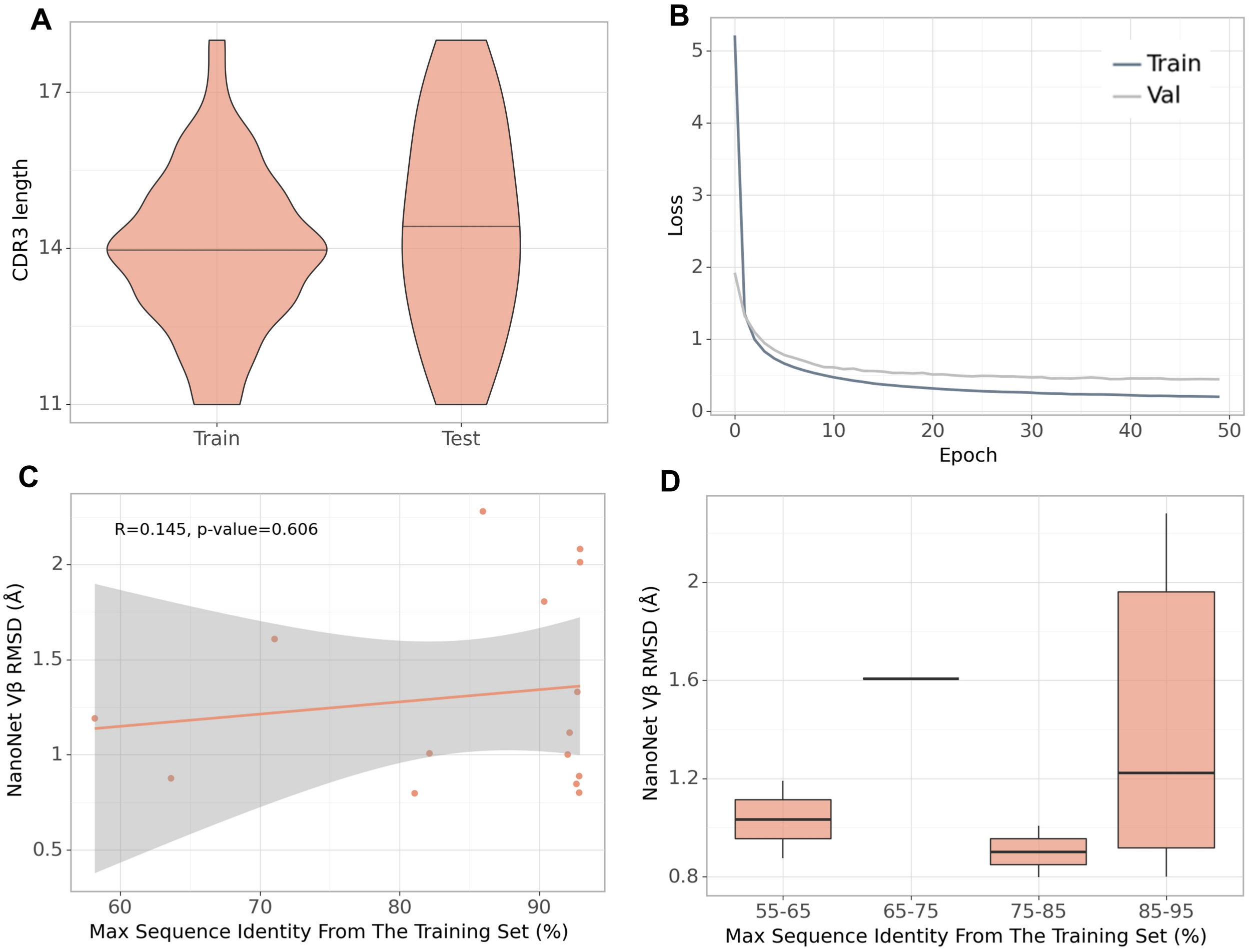

Fig. S2. TCR β chain test set. **(A)** CDR3 lengths of the training and test sets, **(B)** Training and validation loss during the training process, **(C)** Test set performance (measured as Vβ RMSD) as a function of maximal sequence identity to the train set, each dot represents a structure from the TCR test set (15 structures), **(D)** Test set performance with boxplots for sequence identity ranges.

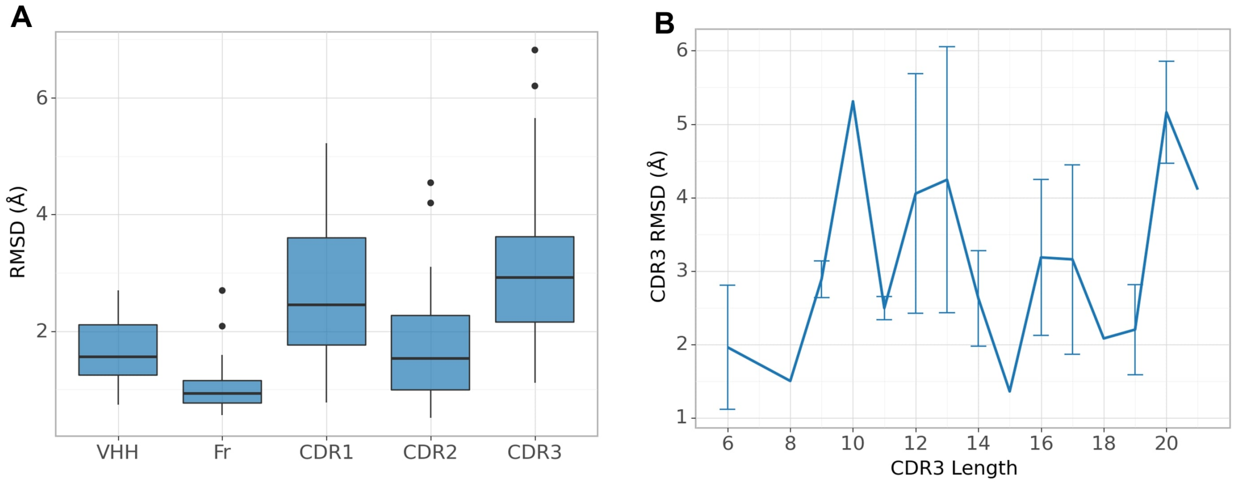

Fig. S3. NanoNet results for the entire Nb test set (44 structures). **(A)** Boxplots of RMSDs of the whole VHH region, framework, CDR1-3 loops, **(B)** RMSD of CDR3 loop as a function of loop length.

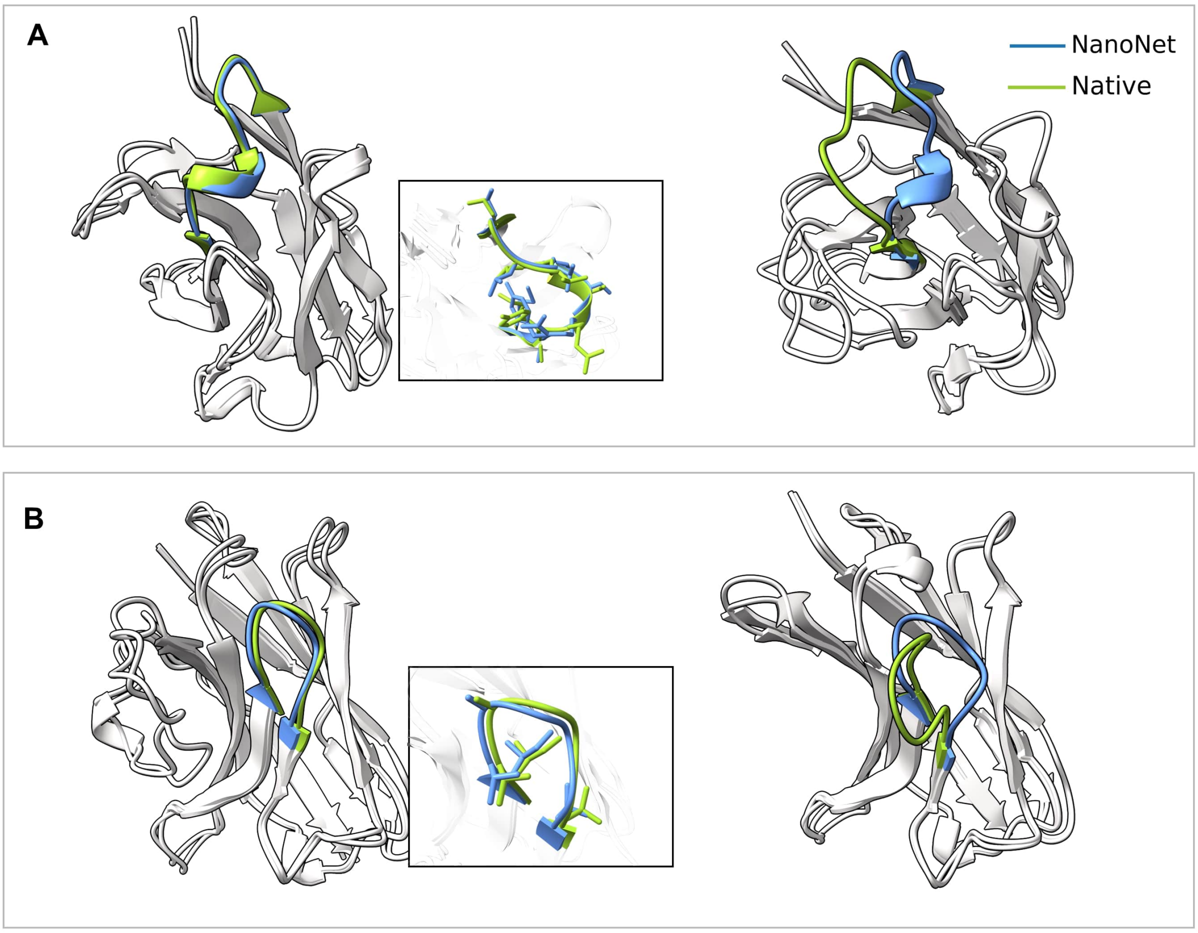

Fig. S4. CDR1 and CDR2 modeling examples of the Nb test set. **(A)** CDR1 modeling: PDB 6xw7 (left) and 6xw4 (right), CDR1 RMSDs 0.78Åand 5.23Årespectively, **(B)** CDR2 modeling: PDB 6z6v (left) and 7mfu (right), CDR2 RMSDs 0.63Åand 4.55Årespectively.

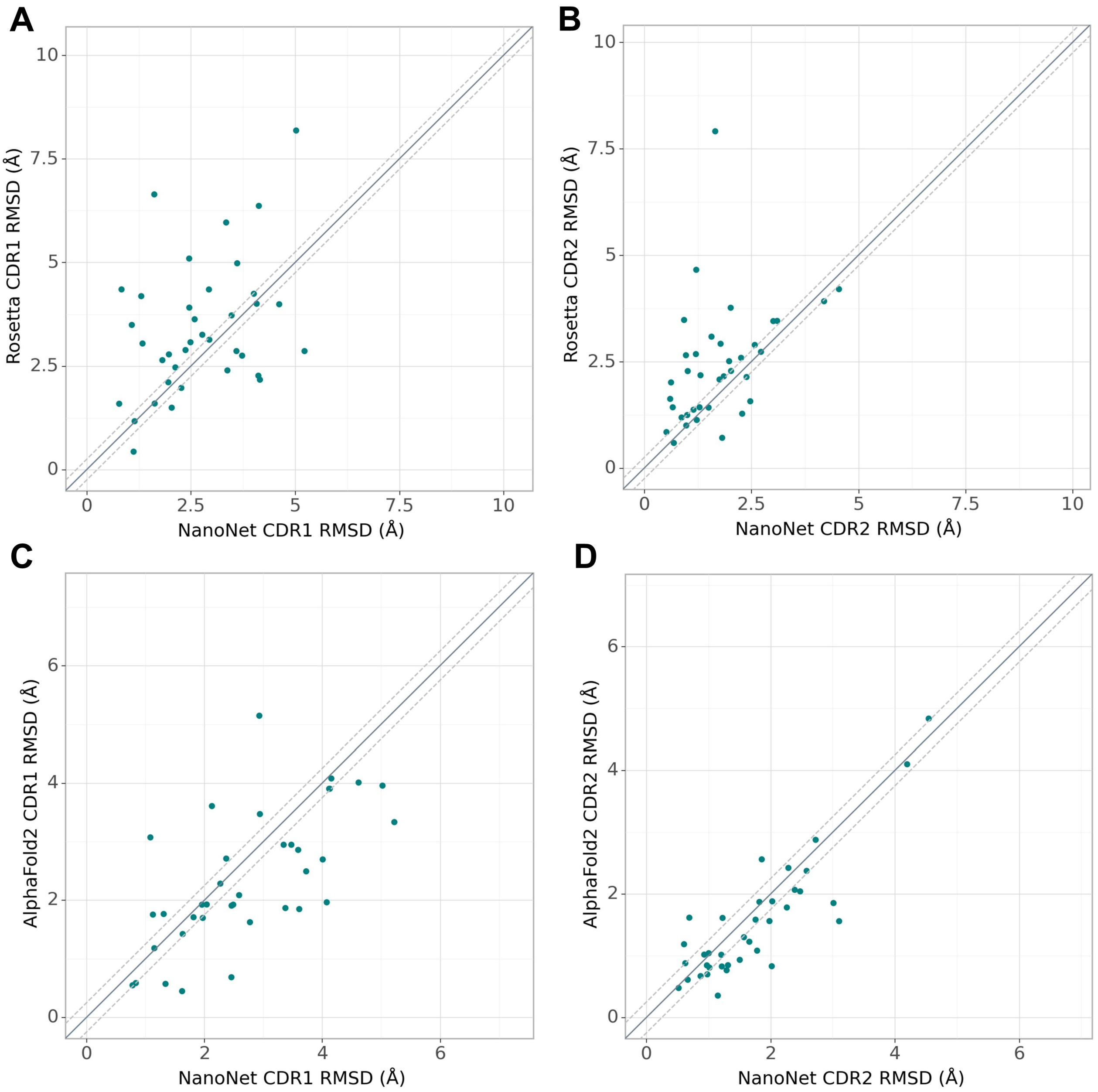

Fig. S5. CDR1 and CDR2 Nb results (37 structures). **(A)** CDR1 loop RMSD for NanoNet vs. RosettaAntibody, each dot represents a structure from the Nb test set. The dotted line corresponds to 0.25Å RMSD, **(B)** CDR2 loop RMSD for NanoNet vs. RosettaAntibody, each dot represents a structure from the Nb test set. The dotted line corresponds to 0.25Å RMSD, **(C)** Same as (A) but for NanoNet vs. AlphaFold2, **(D)** Same as (B) but for NanoNet vs. AlphaFold2

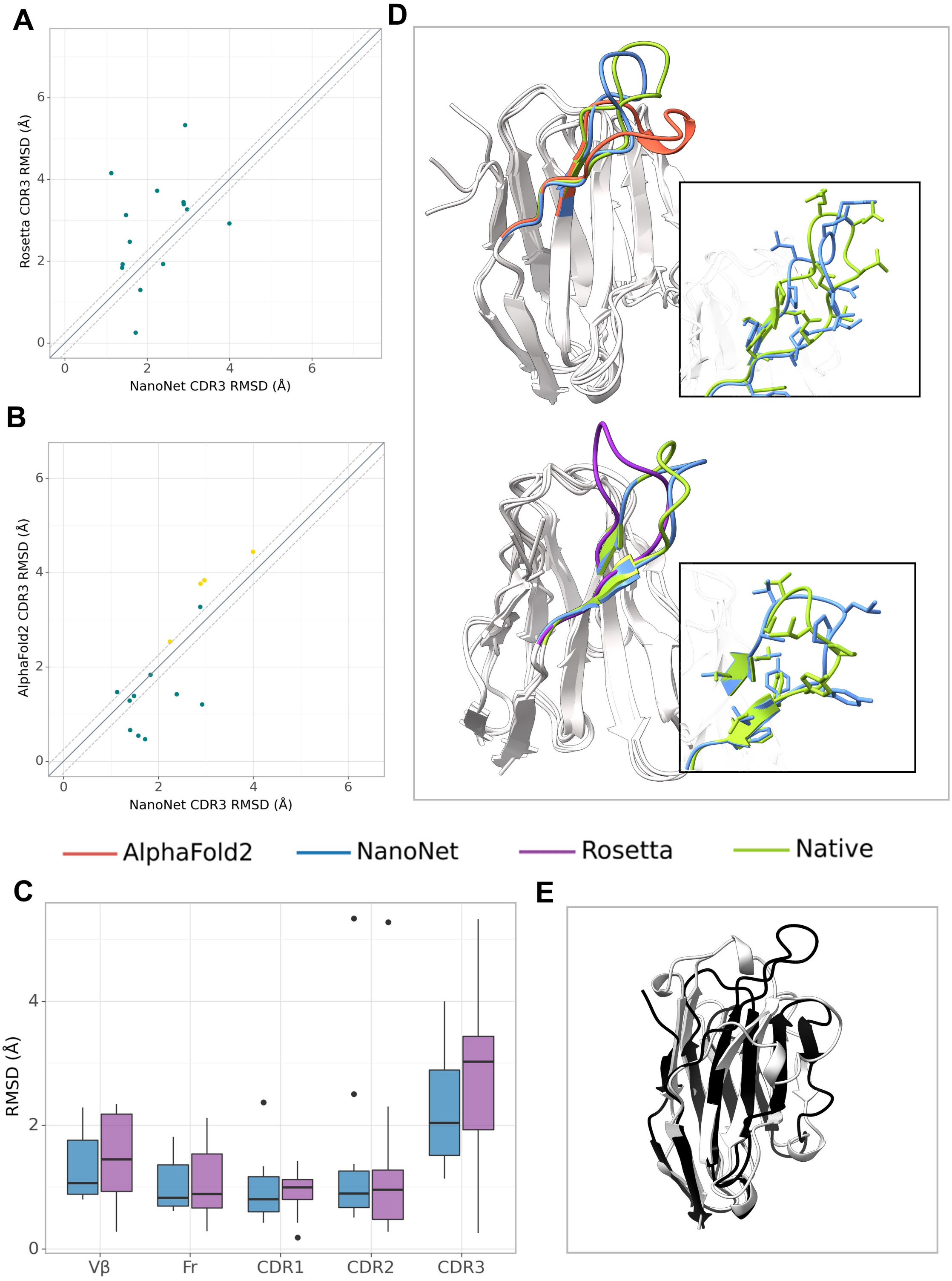

Fig. S6. TCR Vβ chain modeling (14 structures). **(A)** CDR3 loop RMSD for NanoNet vs. Rosetta TCRmodel, each dot represents a structure from the TCR test set. The dotted line corresponds to 0.25 A RMSD, **(B)** Same as (A) but for NanoNet vs. AlphaFold2, structures not in AlphaFold training set are highlighted in yellow. **(C)** Boxplots of RMSDs of the whole Vβ region, framework, CDR1-3 loops for the test set TCRs, **(D)** Test set examples of modeled structures by NanoNet (blue), Rosetta TCRmodel (purple), AlphaFold2 (red) vs. experimental (green): PDB 6ovn (top) - CDR3 RMSD 2.89Å, 3.76Å for NanoNet and AlphaFold2, respectively and 6fr8 (bottom) - CDR3 RMSD 1.49Å ,3.12Å for NanoNet and Rosetta TCRmodel, respectively, **(E)** TCR Vβ (PDB 6r2l, black) aligned to an Nb (PDB 6uht, white).
